# Molecular Dynamics Simulation of Transmembrane Transport of Chloride Ions in Mutants of Channelrhodopsin

**DOI:** 10.3390/biom9120852

**Published:** 2019-12-10

**Authors:** Wenying Zhang, Ting Yang, Shuangyan Zhou, Jie Cheng, Shuai Yuan, Glenn V. Lo, Yusheng Dou

**Affiliations:** 1 Chongqing Key Laboratory of Big Data for Bio Intelligence, Chongqing University of Posts and Telecommunications, Chongqing 40065, China; zhangwenying@cqupt.edu.cn (W.Z.); s170501012@stu.cqupt.edu.cn (T.Y.); zhousy@cqupt.edu.cn (S.Z.); s180501017@stu.cqupt.edu.cn (J.C.); 2 Department of Chemistry and Physical Sciences, Nicholls State University, P.O. Box 2022, Thibodaux, LA 70310, USA; glenn.lo@nicholls.edu (G.V.L.); yusheng.dou@nicholls.edu (Y.D.)

**Keywords:** channelrhodopsin, mutants, cation channel, ion selectivity, molecular dynamics

## Abstract

Channelrhodopsins (ChRs) are light-gated transmembrane cation channels which are widely used for optogenetic technology. Replacing glutamate located at the central gate of the ion channel with positively charged amino acid residues will reverse ion selectivity and allow anion conduction. The structures and properties of the ion channel, the transport of chloride, and potential of mean force (PMF) of the chimera protein (C1C2) and its mutants, EK-TC, ER-TC and iChloC, were investigated by molecular dynamics simulation. The results show that the five-fold mutation in E122Q-E129R-E140S-D195N-T198C (iChloC) increases the flexibility of the transmembrane channel protein better than the double mutations in EK-TC and ER-TC, and results in an expanded ion channel pore size and decreased steric resistance. The iChloC mutant was also found to have a higher affinity for chloride ions and, based on surface electrostatic potential analysis, provides a favorable electrostatic environment for anion conduction. The PMF free energy curves revealed that high affinity Cl^−^ binding sites are generated near the central gate of the three mutant proteins. The energy barriers for the EK-TC and ER-TC were found to be much higher than that of iChloC. The results suggest that the transmembrane ion channel of iChloC protein is better at facilitating the capture and transport of chloride ions.

## 1. Introduction

Channelrhodopsins (ChRs) are non-selective, light-gated cation channels that act as photoreceptor in microalgae and are widely used to activate specific groups of neurons in the brain [1,2,3]. In the dark adaptation state, the ion channel of ChRs are closed. The chromophores of ChRs, retinal molecules, undergo photo-induced trans-cis isomerization, which initiates the photocycle reaction and leads to the opening of ion channels [4,5,6,7]. ChRs can non-selectively conduct monovalent cations such as Na^+^, K^+^, H^+^ and divalent cations such as Ca^2+^. The cation flow into cell will cause depolarization of neuron and lead to an excited neural stimulation. However, for wild-type (WT) ChRs, some physiological studies, such as neural circuit, learning and memory, and movement disorders, cannot be carried out because of the relatively low ion conductivity. Therefore, it is necessary to design and optimize mutants of ChRs to obtain better optogenetic actuators [8].

Mutants of ChRs with different physiological activities can be obtained by modifying their native molecular structures. The mutants have different activation spectra so that a broader range of wavelength can be used to activate the mutational proteins and make them more extensive useful for research on neural regulation [9,10]. In particular, the cation channel of ChRs can be changed into an anion channel by modifying some residues of the WT-ChRs protein. As an opposite kind of optogenetics tool, the inhibiting stimulation induced by anion influent into neuronal cells have made considerable progress in recent years [11,12,13]. In 2014, anion channelrhodopsins (ACRs) were designed [14,15]. The protein can rapidly inhibit the action potential and show the kinetic characteristics of stable inhibition, which is more durable than the light pulse, and the inhibited cells are more sensitive to light. Subsequently, natural ACRs (GtACR1 and GtACR2) were extracted from chlorophyll algae [16]. The designed ACRs have been developed further [17,18,19], and additional natural ACRs have been found by genome mining [20,21,22]. In 2015, the first demonstration of ACRs as inhibitory optogenetic tools that could successfully modulate animal behavior (with a designed ACR named iC++ [17]). Both ACR classes have been widely applied in mice, flies and fish [17,18,23,24,25].

Despite advances in the optogenetics of inhibition, ion binding sites in anion conduction have not been fully identified, and the precise mechanism of ion selectivity and conductivity remains elusive. Electrophysiological investigation [26,27] showed that negatively charged glutamate and aspartate are present among the ChRs transmembrane helixes (TM) TM1, TM2, TM3, and TM7. For example, the crystal structure [26] of chimeric proteins (C1C2) of ChR1 and ChR2 shows that there are seven glutamic acids orientated in the ionic transport pathway of C1C2, which can transport H^+^ and other cations when the channel is opened. It is suggested that the cation selectivity of ChRs is caused by the negative electrostatic potential surface around the channel hole. In the electrostatic surface model, it is hypothesized that the replacement of these residues in or near the pores may reverse channel polarity, resulting in the formation of anion channels.

In this article, we analyzed the precise geometric structure of channel selective filters of several anion-conducting ChRs by molecular dynamics simulation (MD). Based on the MD trajectories, steered molecular dynamics (SMD) simulation, and umbrella sampling analysis were carried out to qualitatively estimate the ion penetration in the anion conduction channel of rhodopsins. This type of study provides insights into the atomic details of ion and channel interactions and open the way for the designing and creating new optogenetic tools.

## 2. Materials and Methods

### 2.1. Simulation-System Preparation

Multiple literature reported [7,28,29,30,31,32,33,34] in recent years, glutamate (E129) in the C1C2 protein is a key amino acid in the center of ion conduction hole and plays an important role in cation selectivity. In order to change the ionic selectivity of the protein, E129 is replaced with alkaline lysine and threonine (T198) is replaced with cysteine to obtain a mutant called EK-TC. If E129 can also be replaced with arginine to obtain a mutant called ER-TC. The mutation could improve membrane targeting of proteins and the enhance binding of retinal in the pocket [10,35]. Three additional mutations can be introduced into the by replacing E122 on the inner side of the channel with glutamine to eliminate the negative charge at that location without changing the residue geometry, replacing E140 outside the channel by serine to reduce cation conduction, and a D195N mutation can be used to extend the open state. This five-fold mutant was E122Q-E129R-E140S-D195N-T198C (iChloC) [18]. Electrophysiological studies [14,30,36] have shown that this combination further enhances chloride selectivity.

Model building on the basis of the C1C2 ground-state structure with PDB ID 3UG9 [26] was performed using the CHARMM-GUI software [37]. For the building of EK-TC and ER-TC models [38]), the E residues of C1C2 was replaced with K and R respectively and T of C1C2 was replaced with C. Furthermore, a five-fold mutation in C1C2-WT was performed (E122Q-E129R-E140S-D195N-T198C, iChloC [18]) for further improving chloride-conducting. The pre-equilibrated 16:0/18:1c9-palmitoyloleyl phosphatidylcholine (POPC) built with a plug of CHARMM-GUI software, was used as a bilayer lipid membrane, and the Membrane Builder module in CHARMM-GUI software was used to optimize the initial structures of each protein, including the addition of side chain and implicit hydrogen atoms and so on. Then the optimized C1C2 structure was embedded in a fully hydrated and balanced POPC membrane, ensuring the protein axis perpendicular to the plane of the lipid membrane. The overlapped phospholipid molecules were discarded to avoid poor van der Waals interactions. The center of the protein was coincided with that of the POPC membrane. Two water boxes, each with a length of 29 Å, were added to the two sides of the system, respectively. Then, 0.15 mol/L of NaCl were used to model the experimental conditions and to ensure the whole system electrically neutral. Finally, the system was simulated using periodic boundary conditions in a simulation box.

### 2.2. Molecular Dynamics Simulations

In this work, the classical molecular dynamics (CMD) simulations were run in NAMD 2.13 [39] software and the CHARMM36 [37] force field and TIP3P water model were applied to describe the transmembrane protein and water molecules, respectively. Simulation process was investigated using a canonical NPT ensemble with periodic boundary conditions, and harmonic potentials applied in x, y, and z directions with a force constant (k) of 10 kcal/mol·Å^2^, respectively, were applied to the backbone C_α_ atoms of the protein to avoid protein inclination. Langevin dynamics was implemented to maintain the temperature of the system at 310 K, with a damping coefficient of 1/ps. Nosé-Hoover Langevin piston method was used to control the system pressure at 1 bar by coupling in XY dimensions with a piston period of 50 fs and a decay of 25 fs. Full electrostatic interactions were treated by the particle mesh Ewald (PME) approach with a grid spacing of less than 1 Å [40]. The cut-off radii of long-range electrostatic and van der Waals interactions were set to be 12 Å, with a smoothing function applied from 10 Å. The bonds containing hydrogen atoms were frozen with SHAKE [41] constrain algorithm and a time step of 2 fs was used in the integration. We used the integration time step of 2 fs, and the trajectories were saved at every 5 ps, with the last 50 ns used for the analysis.

### 2.3. Steered Molecular Dynamics

Although advances in computer technology have extended the time dimensions of dynamic simulations, the time scales for ion permeation through ion channels range from milliseconds to seconds [5,6]. However, the CMD easily lead to the sampling falling into the energy minimum in the conformational space, and it is difficult to realize the process of ion transmembrane conduction. In SMD simulations, a time-dependent external force is applied to the ion to move along the channel from the protein, which cannot usually be achieved by standard MD simulation. For these, SMD was adopted in this work [42,43,44,45,46] and an imaginary external force was artificially applied to Cl^−^ in the simulation so that allows the ion to move along the channel. During the transition, we can calculate the exerted force as well as the external work performed on the system.

All the SMD simulations were performed using the PLUMED [47] plugin integrated in the NAMD 2.13 [39] MD code. Since we were interested in the information about the process of ion transmembrane conduction, the natural choice of the pulling variable was the reaction coordinate of ion, and SMD simulations were carried out as described with constant-velocity [48]. The initial structure in constant-velocity SMD is a representative configuration extracted from cluster analysis. To enable it to move along the direction of the channel, Cl^−^ was initially placed at the entrance of channel and was then pulled in the Z-direction (parallel to the channel axis) from the mouth to the center of the channel at constant velocities, but no restraint was imposed in the X and Y directions. Here, reaction coordinate was defined as the separation between Cl^−^ and the center of the channel, ranging from −18 Å to 17 Å. In order to avoid any distortions of the protein as a consequence of pulling, the Hamiltonian limit of 5 kcal/mol·Å^−2^ was added to the amino acid residues surrounding the seven helices of the protein. The pulling parameters were adopted from Yang et al. [49]. In particular, the spring constant was set to the value of 4 kcal/mol·Å^−2^ to meet the requirements of hard spring and the pulling velocity to 0.0001 Å ps^−1^ to ensure a reversible pulling. Other parameters for SMD simulations are consistent with MD. The time length for each simulation was 360 ns, which was sufficient to observe the entire ion to move along channel process.

### 2.4. Determining the Potential of Mean Force (PMF) with Umbrella Sampling

The potential of mean force of a single Cl^−^ along the channel axis was obtained by employing the Umbrella Sampling (US) method [50,51]. Umbrella Sampling consists of running separate “windows” of the reaction coordinate simultaneously. The change in free energy in each window can be calculated from the sampled distribution of the system along the reaction coordinate. The windows are then combined by methods of the weighted histogram analysis method. Thus, the umbrella sampling simulation could find the equilibrium state in a set of umbrella sampling windows and obtain relatively accurate potential of mean force (PMF) curve by reweighting constant biasing potential well along a reaction coordinate.

The reaction coordinate used for the umbrella sampling simulations was the same reaction coordinate used for the SMD simulations, i.e., the z distance along the channel axis, ranging from −18 Å to 17 Å. With the purpose of enhancing the computational efficiency of the Umbrella Sampling algorithm, the whole span of the reaction coordinate was subdivided into 36 equally spaced windows in the z direction, each with a width of 1 Å. and each window was simulated independently. First, a SMD simulation was carried out to pull Cl^−^ moving through the whole range of reaction coordinate. Then a separate US simulation for each window was performed with the initial structure created from the above SMD trajectory. A harmonic potential with force constant of 2.5 kcal/mol·Å is applied to maintain the respective distance in the z direction for each window, and each window was further divided into 50 bins with a width of 0.1 Å, which was sufficiently small to generate a smooth PMF profile. Finally, we used the Weighted Histogram Analysis Method (WHAM) [52] to construct the PMF based on these umbrella sampling simulation trajectories.

## 3. Results

### 3.1. Water Distributions

**Figure 1:**
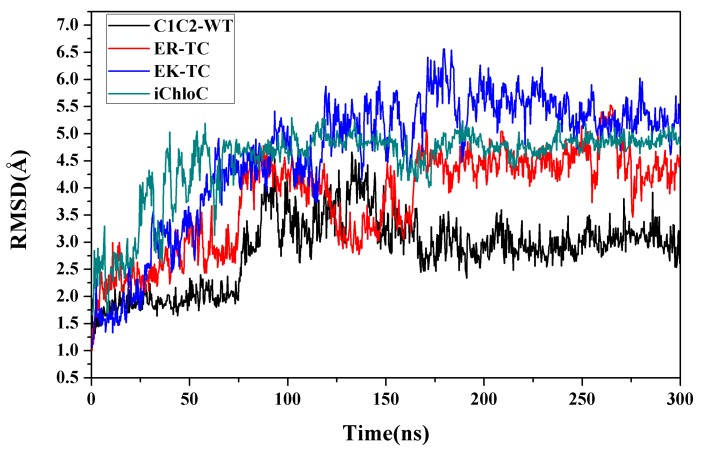
The root mean square difference (RMSD) values of chimeric proteins (C1C2)-wild-type (WT) and mutants EK-TC, ER-TC, and E122Q-E129R-E140S-D195N-T198C (iChloC) over a 300 ns MD simulation.

CMD simulations were performed on wild-type C1C2 (C1C2-WT) and mutants mentioned above respectively to explore the formations of ion channels. In C1C2-WT, protonated E129 forms hydrogen bonds with N297 and S102, respectively. Simultaneously, residues E162 and D292 are deprotonated. After a 300 ns MD simulation, the value of the root mean square difference (RMSD) of the transmembrane helix of each system tended to be stable, indicating that the protein structure tended to be stable and the system had converged, as shown in Figure 1.

**Figure 2:**
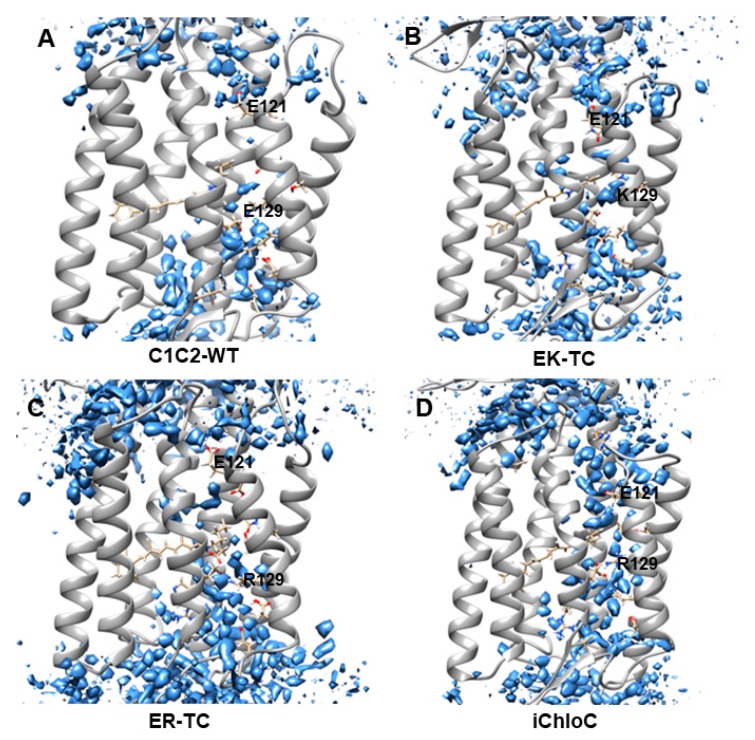
Water distributions of (**A**) C1C2-WT, (**B**) EK-TC, (**C**) ER-TC and (**D**) iChloC of the last 50 ns after molecular dynamics (MD) simulation trajectory equilibrium. The water molecules are shown in blue.

Recent research found water plays an important role in the opening of ion channels in ChR2 [6]. In the early stage of formation of ion channel, outside water flows into cell to form a “pre-opened” channel, then the “pre-opened” channel continues to extend and create ionic conduction states [6]. The water distributions were calculated with grid water density as shown in Figure 2. The water distribution around E129 in C1C2-WT was found to be discontinuous, consistent with the water density in the crystal structure of C1C2 [26], suggesting that the ion channel in natural C1C2 protein is closed. The results of electrophysiological and spectroscopy experiments [26,28,31,32,33,34,53] indicate that E129 participates in the formation of the central gate (CG), and acts as a hydrophobic barrier in the dark state to prevent water from entering the inner vestibule between the central gate and intracellular gate (ICG). After photoactivation of the retinal chromophore, these barriers undergo substantial conformational changes and convert the C1C2 protein to a “pre-opened” state. 

**Figure 3:**
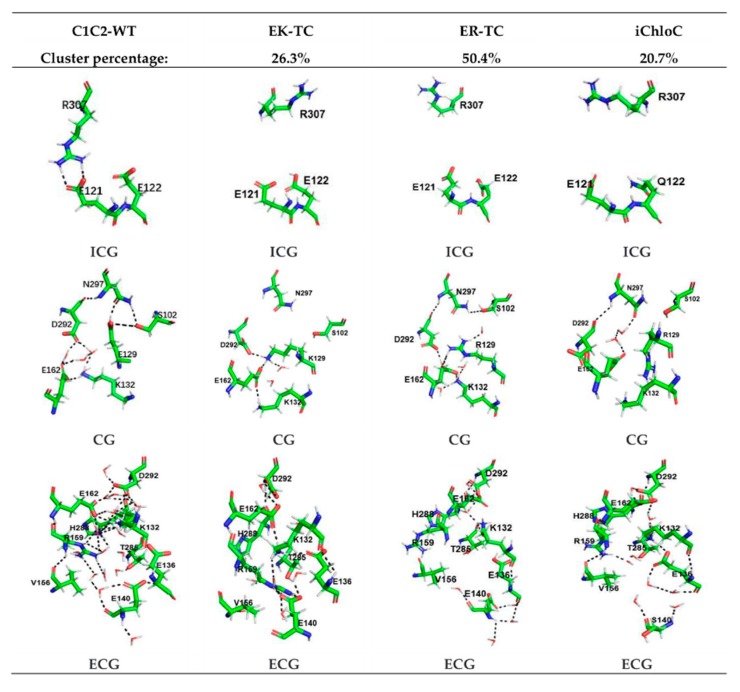
Cluster analysis of EK-TC, ER-TC, and iChloC. The first column shows the stable conformation of intracellular gate (ICG), central gate (CG), and extracellular gate (ECG) after C1C2-WT trajectory equilibrium, Columns 2–4 show the representative conformations of the first class of ICG, CG, and ECG in the EK-TC, ER-TC, and iChloC clustering results, respectively. The percentages of the first class are also shown in the figure.

Figure 2B,C show that fewer water molecules enter the inner vestibule in EK-TC and ER-TC mutants compared with the wild type. The limited number of immigratory water molecules is not enough to be considered as having a continuous distribution in the channel for the water in the mutants EK-TC and ER-TC. However, it is certain that the distribution of water molecules in the mutant ER-TC is significantly different from that in the mutant EK-TC. Compared with E129K, E129R significantly change the structure of extracellular gate (as seen in Figure 3) and lead to more water molecules entering outer vestibule, as shown in Figure 2B,C. So we believe that mutating E129 into arginine may be more conducive to the formation of anion channels. Further mutation (iChloC, Figure 2D) shows that more water molecules enter the inner vestibule, and a continuous water distribution between the two vestibules is obtained. This suggests that the additional mutation in iChloC is favors the influx of more water consistent with the formation of a pre-opening channel. These findings are consistent with the results in the photocurrent experiment [18,38].

### 3.2. Structure Changes in Mutants

In order to intuitively display the conformational differences of different mutants systems, the K-means algorithm in the MMTSB toolset [36] was used for the cluster analysis of simulation trajectories of the mutants EK-TC, ER-TC, and iChloC. 

In Figure 3, the stable conformations of the ICG, CG, and extracellular gate (ECG) of C1C2-WT are separately presented. It was observed that, as in the crystal structure reported in the literature [26], the protein channel is mainly blocked by two constrictions—the central gate and the intracellular gate. The CG in the center of the channel is composed of residues S102, E129, D292, K132, and N297. The key amino acid E129 forms hydrogen bonds with N297 and S102, respectively. D292, E162, and K132 are also connected to each other intermediated by water molecules to form a hydrogen bond network, which blocks the central gate and closes the channel. The ICG of the C1C2-WT protein is composed of residues E122, E121, and N297. The formation of double hydrogen bonds between residues R307 and E121 also suggests the inside of the ion channel is blocked. In addition, we found that the channel of extracellular part is also discontinuous. There are several water molecules in the ECG, which consists of residues V156, E140, R159, E136, and H288. The water molecules and the key residue R159 play a pivotal role in the formation of hydrogen bonds network among the ECG and its adjacent amino acids, which tightly constrains TM2, TM3, and TM7 and blocks the ion channel. Moreover, Figure 3 shows that the hydrogen bond network formed in the ECG is very complicated and extends all the way to the CG, so that it can be considered that the ECG and the CG are closely related.

Compared with C1C2-WT, the ICG, CG, and ECG of the mutant are significantly different. In the ICG, residue R307 in the mutants are flipped, which led to the destruction of the double hydrogen bond originally formed with E121. Furthermore, the E121 and E122 of the three mutants all tilted slightly downward. In particular, in iChloC, where E122 was mutated to glutamine (Q), the conformation of Q122 is flipped, leaving a larger cavity for the intracellular gate. This phenomenon indicates that E122Q is beneficial to the opening of the intracellular gate. In CG, the structures of K129 and R129 were flipped after mutating E129 into lysine and arginine, making the original hydrogen bond with N297 and S102 disappear. However, according to the results of water distribution, water molecules did not penetrate the central gate well in the EK-TC and ER-TC mutants (Figure 2B,C). By observing the central gates of the two mutants, it was found that both the lysine and the arginine after the mutation were bulky residues, and because of the limited space of the central gate, the conformations of K129 and R129 were not changed in the direction towards the inside of the helix, thus blocking the central gate. On the other hand, the conformation of R129 in iChloC is flipped, opening the central gate and allowing water molecules to penetrate well through the central gate (Figure 2D). By observing ECG, it was found that the hydrogen bond complexity in the ECG conformation in ER-TC and iChloC was significantly reduced compared to C1C2-WT. This is due to the flipping of the key amino acid R159, which destroys the hydrogen bond network in the ECG, creating a large cavity in the extracellular portion. This suggests that the mutation of E129 into arginine is more favorable for the opening of the extracellular channel. We also found that in iChloC, where E140 in the extracellular part was mutated into serine, the cavity of the extracellular channel is more enlarged compared with ER-TC. Due to the destruction of the ECG hydrogen bond network of the iChloC mutant, the connection between TM2, TM3, and TM7 is broken, TM2 is easier to tilt outward, and more space is reserved for the central gate. This explains why only the R129 conformation at the central gate of the iChloC mutant has changed, allowing water molecules to penetrate well through the central gate.

The results of structural analysis show that the three mutants of C1C2 showed significant changes in the three blockages of the ion channel compared to ChR2-WT. These changes are consistent with the expected trend of ion channel formation in the literature [18,38]. The conformational change of the mutant iChloC is towards the direction more favorable for the opening of the channel. Due to the rearrangement of the hydrogen bonding network, a continuous water distribution is formed between the two vestibules (Figure 2D).

### 3.3. The Electrostatic Potential Surface of the Channel

**Figure 4:**
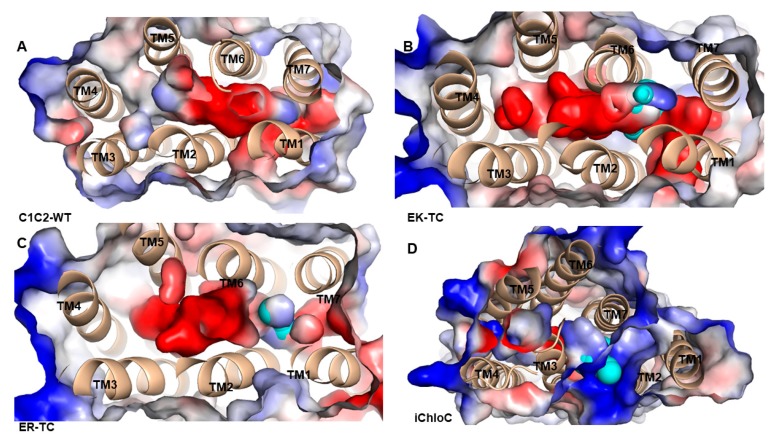
Electrostatic potential surface of of (**A**) C1C2-WT, (**B**) EK-TC, (**C**) ER-TC and (**D**) iChloC. The pore surface is calculated by CAVER3.0.1 program, represented by cyan. The electrostatic potential is calculated by PyMOL. The red is the negative potential and the blue is the positive potential.

The electrostatic surface potential of C1C2-WT and its mutants were calculated on the pore surface (Figure 4). In C1C2-WT (Figure 4A), an electronegative hole, which consists of negatively charged residues including E122, E129, E136, E140, E162, and D292 was formed among TM1, TM2, TM3, and TM7. These residues contribute to the formation of electronegative surface suitable for cation selectivity. As shown in Figure 4B,C, in the mutants EK-TC and ER-TC, the electrostatic potential formed a small amount of positive potential surface between TM2 and TM7 compared to the wild type. The electrostatic potential around the iChloC pore indicates that the ion channel of the mutant is also electrically positive (Figure 4D). These positive electrostatic potentials make the protein interior accessible to chloride ion.

Since most of the negatively charged residues are derived from TM2, we suggest that the ionic conductance and selectivity of C1C2-WT are primarily determined by TM2. The E122 in TM2 is a key residue of the ICG that stretches the negatively charged carboxylic acid group into the pores and leads to the formation of a potential barrier for anion diffusion. Similarly, negatively charged E140 located at the entrance of the ECG possibly hinders the permeation of anions. Therefore, in mutant iChloC, where E122, E129, and E140 on TM2 were replaced by glutamine, arginine, and serine, respectively, the electronegativity surface around the pores was reversed. It suggests that these residues provide a suitable electrostatic environment for anion conduction in iChloC and also explains the experimental result that the photo-induced membrane depolarization of the iChloC mutant is greatly attenuated [18].

### 3.4. Potential of Mean Force from Umbrella Sampling

**Figure 5:**
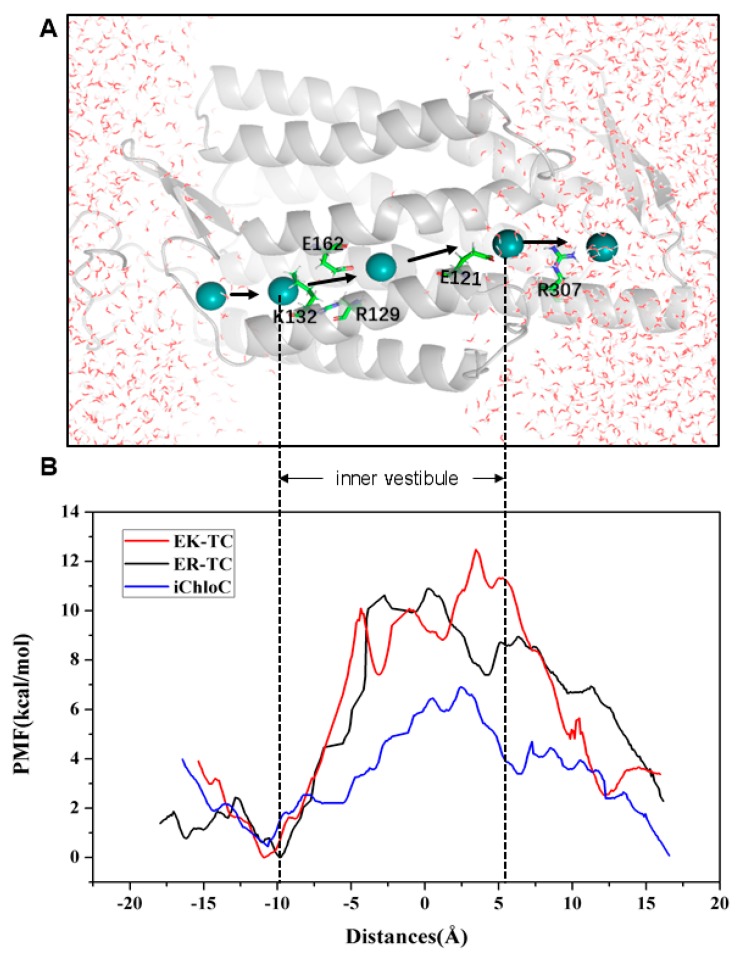
(**A**) Schematic diagram of the simulated trajectory of ion going through the channel. Cl^−^ is represented by a blue ball. (**B**) Potential of mean force (PMF) reconstructed using umbrella sampling for Cl^−^ permeation across ion channel in mutants.

To further understand the selectivity of ion permeation, we adopted SMD simulation to move Cl^−^ through the ion channels of three mutants (Figure 5A), and further estimated the energy changes of ion transmembrane transport of related ions by using the umbrella sampling [42] method. The constructed PMF of Cl^−^ through channels of three mutants was shown in Figure 5B. It can be seen from the PMF curve of the EK-TC mutant that it is an energy reduction process for Cl^−^ entering the ion channel. The energy is gradually reduced from 3.90 kcal/mol to 0 kcal/mol, and an energy minimum is reached at K132 of the CG (at about −9 Å). Obviously, there is electrostatic attraction between negatively charged Cl^−^ and positive charged lysine. Hence the outer vestibular part of the ion channel has the ability to capture and enrich chloride ions. Therefore, we suggest that K132 is the binding site of Cl^−^ in the ion channel. Subsequently, Cl^−^ encounters a bottleneck with energy barrier of about 10 kcal/mol in the ion channel. Clearly, as reflected from the PMF, energy increases as Cl^−^ passes through the inner vestibule between the CG and the ICG. After Cl^−^ ions pass through the inner vestibule and ICG (about 7.5 Å), it gradually moves away from ion channel and solvated.

The PMF curve of ER-TC and EK-TC are very similar, with a potential energy wells at the same binding site, K132. However, there are differences. In ER-TC, as chloride passes through the entrance of ion channel, energy does not decrease as much (3.90 kcal/mol to 2.43 kcal/mol). This can be explained by the E129R mutation causing the R159 at ECG to flip. This destroys the hydrogen bond networks, which makes it easier for ER-TC mutant to capture and enrich Cl^−^ in the outer vestibule. The energy barrier of Cl^−^ in the inner vestibule between the CG and the ICG in ER-TC mutant about 0.59 kcal/mol lower, on average, compared to EK-TC. This is also consistent with E129R in ER-TC being more favorable for the formation of anion channels than E129K mutation in EK-TC.

A significant difference can be noted in the PMF curve of iChloC compared to ER-TC and EK-TC. The energy barriers for Cl^−^ pass through CG is much smaller in iChloC and there is no energy rise in the inner vestibule between the CG and ICG. Therefore, it is easier for Cl^−^ to be traverse the ion channel in iChloC. This result is consistent with the conformational changes as discussed above as well as with the experimental results [18].

Generally, it is useful to understand the channel function of the hydrophobic region in the biological ion channel by analyzing the energy barrier for ions passing through the channel. This can provide a theoretical basis for designing novel light-controlled ion channel proteins. For example, Cl^−^ more easily passes through the CG of iChloC compared to the mutant EK-TC and ER-TC, which means that in addition to local electrostatic interaction in the biological ion channel, the pore size of the hydrophobic cavity also an important factor to consider for ion selectivity.

## 4. Conclusions

As regulator of neurons, understanding ion channels has always been one of the important goals of optogenetics. In this article, molecular simulation and umbrella sampling methods were used to study the molecular mechanism of the transport of Cl^−^ through ChRs. The results show that point mutations of E129R and E140S increase the structural flexibility of the transmembrane channel protein of the five-fold mutant (iChloC). the outward tilt of TM2 increases the pore size of ion channel and lowers the steric hindrance. From the electrostatic potential analysis, it was found that mutating the negatively charged amino acids E129, E122, and E140 on TM2 into positively-charged or uncharged amino acids in iChloC contributes to the formation of electropositive surface leading to anionic selectivity in and around the pores. The formation of electropositive surface provides a highly favorable electrostatic environment for anion conduction in the iChloC mutant. By analyzing the change of PMF curve obtained from umbrella sampling, it can be hypothesized that Cl^−^ has a high affinity binding site and energy potential well at K132 (Figure 4), which hinders the transport of Cl^−^ through the CG of the channel. In addition, PMF curves suggest that the energy barriers for the mutant EK-TC and ER-TC in the CG are much higher than those for iChloC. The five-fold mutation in iChloC significantly enhances the ability of the transmembrane protein to capture and enrich chloride ions. In this work, the structure-oriented analysis of multiple mutants led us to have a deeper understanding for the ChR pores. We can conclude that protein flexibility, Cl^−^ affinity, and the hydrogen bonding network are important factors in the formation of cavities and the enhancement of anion selectivity and conductance of the mutant transmembrane protein. Taking everything into consideration, this study provides a theoretical basis for the formation mechanism and ion permeation mechanism of ion channels, and provides a way for rational protein engineering of channelrhodopsin ion pores.
